# CircZFR functions as a sponge of miR-578 to promote breast cancer progression by regulating HIF1A expression

**DOI:** 10.1186/s12935-020-01492-5

**Published:** 2020-08-18

**Authors:** Zhuo Chen, Fang Wang, Youyi Xiong, Nan Wang, Yuanting Gu, Xinguang Qiu

**Affiliations:** 1 Department of Breast Surgery, The First Affiliated Hospital of Zhengzhou University, Zhengzhou, 450000 China; 2 Department of Thyroid Surgery, The First Affiliated Hospital of Zhengzhou University, No.1 Jianshe East Road, Erqi District, Zhengzhou, 450000 Henan China

**Keywords:** Breast cancer (BC), circZFR, miR-578, HIF1A, Malignant progression

## Abstract

**Background:**

Breast cancer (BC) is the most common malignancy among women. Emerging studies have demonstrated that circular RNA (circRNA) zinc finger RNA binding protein (circZFR) serves as a crucial regulator in many human cancers. However, the role and mechanism of circZFR in BC tumorigenesis remain unclear.

**Methods:**

The levels of circZFR, miR-578 and hypoxia-inducible factor 1α (HIF1A) were detected by quantitative real-time polymerase chain reaction (qRT-PCR) or western blot. Cell viability, colony formation, apoptosis, migration and invasion capacities in vitro were determined by using the Cell Counting Kit-8 (CCK-8), standard colony formation, flow cytometry and transwell assays, respectively. Glucose uptake, lactate product and adenosine triphosphate (ATP) levels of cells in vitro were measured using the commercial human assay kits. Targeted relationships among circZFR, miR-578 and HIF1A in BC cell lines were verified by dual-luciferase reporter and RNA pulldown assays. Animal studies were performed to assess the effect of circZFR on tumor growth in vivo.

**Results:**

Our data indicated that circZFR was overexpressed in BC tissues and cells, and the increased circZFR level predicted poor prognosis of BC patients. CircZFR silencing or miR-578 overexpression repressed BC cell viability, colony formation, migration, invasion, and glycolysis and enhanced cell apoptosis in vitro. CircZFR silencing also hampered tumor growth in vivo. Mechanistically, circZFR acted as a sponge of miR-578, and circZFR silencing hindered BC cell malignant behaviors by miR-578. HIF1A was a functional target of miR-578 in regulating BC cell viability, colony formation, migration, invasion, glycolysis and apoptosis in vitro. Furthermore, circZFR modulated HIF1A expression through sponging miR-578.

**Conclusion:**

Our findings first identified that the silencing of circZFR suppressed BC malignant progression in vitro via the regulation of the miR-578/HIF1A axis, providing evidence for the crucial involvement of circZFR in BC pathogenesis.

## Background

Breast cancer (BC) remains the most commonly diagnosed cancer and the leading cause of cancer-associated death among females in 2018 [1]. Although the therapeutic methods have greatly improved over the past two decades, effective treatment against metastatic BC is still limited [2, 3]. Therefore, a deeper understanding of what drives this disease is the first step to design innovative interventions.

Circular RNAs (circRNAs) are covalently closed, endogenous RNAs that have crucial non-coding functions in human physiologic and pathologic processes [4]. Work in biological functions has demonstrated the roles of circRNAs as microRNA (miRNA) sponges [5]. Dysregulation of circRNAs has recently implicated in the pathogenesis of BC [6]. For example, Yuan et al. uncovered that hsa_circ_0068033, a down-regulated circRNA, exerted a repressive impact on BC malignant progression via sequestering miR-659 [7]. Cao and colleagues demonstrated that hsa_circ_0087784 functioned as a potential promoter in BC development through sponging miR-487a [8]. Xu et al. reported that circTADA2As attenuated BC progression and metastasis by the regulation of miR-203a-3p [9]. Moreover, hsa_circ_001783 and circABCB10 were reported as oncogenic regulators in BC through functioning as specific miRNA sponges [10, 11]. As for circRNA zinc finger RNA binding protein (circZFR, hsa_circ_0072088), it has been identified as an oncogenic modulator in many human cancers, such as renal carcinoma, bladder cancer and non-small cell lung cancer [12–14]. Nevertheless, the biological roles of circZFR in BC tumorigenesis remain largely unknown.

MiRNAs modulate gene expression but are frequently dysregulated in human cancers, including BC [15]. Danza et al. reported that miR-578 was underexpressed in BRCA-BC and it regulated tumor angiogenesis [16]. However, the precise function of miR-578 in BC progression is still undefined.

Here, we undertook to examine the biological effect of circZFR in the malignant progression of BC in vitro and in vivo. We identified that circZFR, a prominently up-regulated circRNA in BC, controlled BC progression in vitro via targeting the miR-578/hypoxia-inducible factor 1α (HIF1A) axis.

## Materials and methods

### Clinical tissues and cells

In this study, we enrolled 70 BC patients admitted to The First Affiliated Hospital of Zhengzhou University from April 2013 to June 2014. The clinicopathological features of these patients were provided in Table 1. Seventy pairs of primary tumor tissues and matched healthy breast tissues were collected and stored at − 80 °C to detect the expression levels of circZFR, miR-578 and HIF1A (Accession: NM_181054.3). These patients were followed-up for at least 60 months and the follow-up information was obtained by telephone calls every 3 months. The study was approved by the Ethics Committee of The First Affiliated Hospital of Zhengzhou University, and written informed consent was provided by all participants.

**Table 1:** Correlation between circZFR expression and the clinicopathological features of BC patients

Characteristics	Number	circZFR expression		*P*
		High	Low	
		35	35	
Age (years)				0.473
< 60	33	15	18	
≥ 60	37	20	17	
Distant metastasis				0.016*
Present	32	11	21	
Absent	38	24	14	
Tumor size (cm)				0.629
≤ 2	30	16	14	
> 2	40	19	21	
TNM stage				0.002**
I, II	37	25	12	
III, IV	33	10	23	
HER-2 status				0.339
Positive	34	15	19	
Negative	36	20	16	
PR status				0.229
Positive	31	13	18	
Negative	39	22	17	
ER status				0.473
Positive	37	17	20	
Negative	33	18	15	

*HER-2* human epidermal growth factor receptor-2, *PR* progesterone receptor, *ER* estrogen receptor

*P < 0.05, **P < 0.01

BC cell lines MCF7 (ATCC^®^HTB-22), BT-549 (ATCC^®^HTB-122), MDA-MB-231 (ATCC^®^HTB-26) and MDA-MB-453 (ATCC^®^HTB-131, American Type Collection Culture, ATCC, Manassas, VA, USA) were cultured in RPMI-1640 medium, supplemented with 10% fetal calf serum (FCS) and 1% antibiotic solution (all from HyClone, Logan, UT, USA). The immortalized MCF10A cell line (ATCC^®^CRL-10317, ATCC) was maintained in Dulbecco’s modified Eagle’s medium/Nutrient Mixture F-12 (DMEM/F-12) with 10% FCS, 20 ng/mL epidermal growth factor, 0.5 μg/mL hydrocortisone and 5 μg/mL insulin (all from HyClone). All cells were cultured in a 5% CO_2_ incubator at 37 °C.

### Quantitative real-time polymerase chain reaction (qRT-PCR)

The expression levels of circZFR, HIF1A and miRNAs were gauged by qRT-PCR. Complementary DNA (cDNA) synthesis was done using total RNA (100 ng) isolated by Isogen (Nippon Gene, Tokyo, Japan) from tissues and cell lines. The levels of circZFR and HIF1A were quantified using the TaqMan Gene Expression Assays with the indicated primers, and mature miRNAs were assayed using the TaqMan MicroRNA Assays with TaqMan-specific primer probes as recommended by the manufacturers (Applied Biosystems, Rotkreuz, Switzerland). qRT-PCR was run in triplicate on the iCycler iQ5 device (Bio-Rad, Munich, Germany) using the PCR conditions as previously reported [17]. Results were normalized to the expression of glyceraldehyde-3-phosphate dehydrogenase (GAPDH) or U6 (internal control) using the 2^−ΔΔCt^ method [17]. Primer sequences for circZFR were: forward, 5′-ATGGTCTGCAGTCCTGTGTG-3′ and reverse, 5′-TGGTGGCATGTTTTGTCATT-3′; for HIF1A were: forward, 5′-TTCCCGACTAGGCCCATTC-3′ and reverse, 5′-CAGGTATTCAAGGTCCCATTTCA-3′; for miR-578 were: forward, 5′-GTGCAGGGTGTTAGGA-3′ and reverse, 5′-GAAGAACACGTCTGGT-3′; for miR-944 were: forward, 5′-GAGTAGGCTACATGTTATTAAA-3′ and reverse, 5′-GTGCAGGGTCCGAGGT-3′; for miR-532-3p were: forward, 5′-ATCCTCCCACACCCAAGG-3′ and reverse, 5′-GTGCAGGGTCCGAGGT-3′; for GAPDH and U6 were described previously [18].

### Lentivirus transduction and transient transfection

CircZFR knockdown in MCF7 and BT-549 cell lines was achieved by the transduction of corresponding lentiviruses expressing three different sequence shRNAs specific to circZFR (sh-circZFR#1 (sh-circZFR), sh-circZFR#2 and sh-circZFR#3, Fulengen, Guangzhou, China), and nontarget shRNA lentiviruses (sh-NC) were used as the negative control. Vector-transduced cells were selected by puromycin (Solarbio, Beijing, China) at a final concentration of 2.5 μg/mL at least 72 h. MiR-578 overexpression and knockdown cell lines were generated using the synthetic miR-578 mimic (30 nM, Ribobio, Guangzhou, China) and inhibitor (anti-miR-578, 30 nM, Ribobio), respectively, with a corresponding nontarget oligonucleotide (miR-NC mimic or anti-NC, Ribobio) as the negative control. To elevate HIF1A expression in BC cell lines, a recombinant overexpressing plasmid for HIF1A (HIF1A, 100 ng, Ribobio) or negative control plasmid (vector, Ribobio) was transiently transfected into cell lines using Lipofectamine 3000 reagent (Invitrogen, Breda, The Netherlands) as per the manufacturers’ protocols. Each experiment was performed in triplicate.

### Cell viability, colony formation and apoptosis assays

sh-circZFR-infected or sh-NC-transduced cell lines were transfected with or without anti-NC or anti-miR-578, and MCF7 and BT-549 cell lines were carried out the indicated transfections, followed by the incubation for 24 h at 37 °C. Cell viability assay was done using the Cell Counting Kit-8 (CCK-8, Abcam, Cambridge, UK) assay as per the manufacturer’s guidance. Cell colony formation was tested using standard colony formation protocols as previously reported [19]. Cell apoptosis measurement was performed by flow cytometry using double-staining with fluorescein isothiocyanate (FITC)-labeled Annexin V and propidium iodide (PI) based on the directions of manufacturers (Invitrogen). 10,000 events were analyzed by a flow cytometer, and the apoptotic cells were determined by calculating the sum of early (Annexin V^+^/PI^−^) and late (Annexin V^+^/PI^+^) apoptotic cells. All experiments were done in triplicate.

### Transwell migration and invasion assays

Cell migration and invasion were detected by the transwell assay using modified Boyden chambers in 24-transwell plates (8 μm pores, Corning, Amsterdam, The Netherlands). Chambers of cell invasion assays consisted of Matrigel-precoated membrane inserts (Corning). After transfection, BC cell lines were seeded into the top chamber of a 24-transwell insert, and medium containing 15% FCS was used as a chemoattractant in the lower chamber. 24 h later, the well was stained with 1% crystal violet (Solarbio), and the number of cells that had migrated or invaded to the basal side of the membrane was counted under a microscope (Nikon, Shinagawa, Tokyo, Japan) at 100× magnification. Each experiment was performed in triplicate.

### Measurement of glucose uptake, lactate product and adenosine triphosphate (ATP) level

The Colorimetric Glucose Uptake Assay Kit, L-Lactate Assay Kit and ATP Assay Kit (all from Abcam) were used to determine the levels of glucose uptake, lactate product and ATP, according to the recommendations of manufacturers. Briefly, the lysates of transfected cells were prepared using the Assay buffer and incubated with standard protocols for the indicated time point, followed by the measurement of the absorbance with a microplate reader (Invitrogen) at OD 412 nm for glucose uptake, 450 nm for lactate product and 570 nm for ATP level. All assays were done in triplicate.

### Animal studies

The xenograft models were constructed to assess the role of circZFR on tumor growth in vivo. Animal studies were implemented in accordance with a protocol approved by the Ethics Committee on Animal Use and Care of the First Affiliated Hospital of Zhengzhou University. Twelve 6–8 weeks BALB/c female mice (Shanghai Animal Laboratory Center, Shanghai, China) were used and randomly divided into two groups (n = 6 per group): sh-NC and sh-circZFR. Approximately 5 × 10^6^ sh-NC-infected or sh-circZFR-transduced BT-549 cell line was subcutaneously injected into the dorsal flank of the mice. One week later, tumor size was measured every week with callipers and tumor volume was calculated using the following formula: volume = 0.5 × length × (width)^2^. All mice were sacrificed at 35 days after implantation, and tumor tissues were collected for weight.

### Bioinformatics, dual-luciferase reporter and RNA pulldown assays

Analysis for the targeted miRNAs of circZFR was performed using the online web CircInteractome (https://circinteractome.nia.nih.gov/index.html) and CircBANK (http://www.circbank.cn/searchCirc.html). The putative targets of miR-578 were predicted by TargetScan v.7 online software (http://www.targetscan.org/vert_71/).

Targeted relationships among circZFR, miR-578 and HIF1A were confirmed by dual-luciferase reporter and RNA pulldown assays. In dual-luciferase assays, the fragment of circZFR containing the miR-578-binding sites and HIF1A 3′UTR were individually cloned into pmirGLO vector (Promega, Madison, WI, USA) to construct corresponding wild-type reporters (circZFR-WT and HIF1A-3′UTR-WT). The TaKaRa MutanBest Kit was used to construct the corresponding mutations (circZFR-MUT and HIF1A-3′UTR-MUT) as per the insturctions of manufacturers (TaKaRa, Dalian, China). Each reporter construct (200 ng) and 30 nM of miR-578 mimic or miR-NC mimic were cotransfected into BC cell lines. Cell line extracts were prepared with RIPA buffer (TaKaRa) 24 h post-transfection, and the ratio of Renilla to firefly luciferase was detected using the Promega Dual-luciferase Assay. In RNA pulldown assays, cell lysates were incubated with the biotin-labeled miR-578 mimic (Bio-miR-578, Ribobio) or nontarget control sequence (Bio-NC, Ribobio) for 4 h at 4 °C before adding to the streptavidin beads (Sigma-Aldrich) for 2 h. The beads were collected, and total RNA was extracted for circZFR quantification. Each experiment was performed in triplicate.

### Western blot for HIF1A expression

Western blot was used to determine the expression of HIF1A using standard protocols. The preparation of cell line extracts was done using RIPA buffer with proteinase inhibitors (Roche, Charente, France). Proteins (50 μg) were resolved on a 10% SDS–polyacrylamide gel, electrophoretically blotted onto nitrocellulose membranes (GE Healthcare, Little Chalfont, UK) and probed with antibody against HIF1A (ab51608, 1 μg/mL, Abcam) or GAPDH (MA5-15738, 0.5 μg/mL, Invitrogen). Following the incubation with horseradish peroxidase-coupled IgG secondary antibody (ab97051, 0.1 μg/mL, Abcam), the signals were visualized by Cheniluminescence (GE Healthcare) as recommended by the manufacturers. All experiments were done in triplicate.

### Statistical analysis

Data were shown as the mean ± standard deviation from at least three independent assays. Pairwise comparisons were done using a two-tailed Student’s *t* test, Mann–Whitney *U* test or analysis of variance (ANOVA) with SPSS version 19.0 software (SPSS, Chicago, IL, USA). For survival analysis, the Kaplan–Meier survival curve and log-rank test were used. Correlations among circZFR, miR-578 and HIF1A expression levels in BC tissues were determined by the Spearman correlation test. All tests were considered statistically significant at *p*-value < 0.05.

## Results

### Overexpression of circZFR predicted poor prognosis of BC patients

**Fig. 1:**
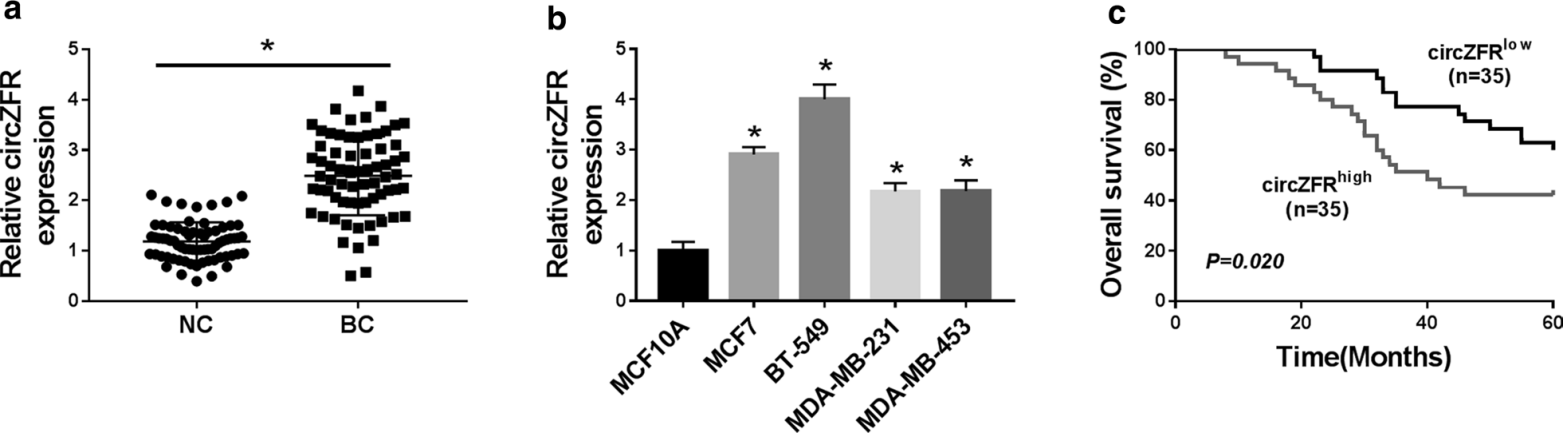
CircZFR was overexpressed in BC and associated with poor prognosis. CircZFR expression by qRT-PCR in 70 pairs of tumor tissues and adjacent normal tissues (**a**), MCF10A, MCF7, BT-549, MDA-MB-231 and MDA-MB-453 cell lines (**b**). **c** Analysis for the overall survival of BC patients in high (n = 35) or low (n = 35) circZFR level group divided by the median of circZFR expression in BC tissues using Kaplan–Meier survival analysis and log-rank test. *P < 0.05

As demonstrated by qRT-PCR, circZFR was significantly up-regulated in BC tissues and cell lines compared with their counterparts (Fig. 1a, b). To determine its clinical relevance, we preliminarily examined the link between circZFR level and the prognosis of BC patients. Kaplan–Meier survival curves showed that the patients in low circZFR level group had a longer survival time than those in high circZFR group (Fig. 1c). Additionally, circZFR expression was remarkably associated with the distant metastasis and TNM stage of these patients (Table 1).

### Silencing of circZFR hindered BC cell viability, colony formation and enhanced apoptosis in vitro and weakened tumor growth in vivo

**Fig. 2:**
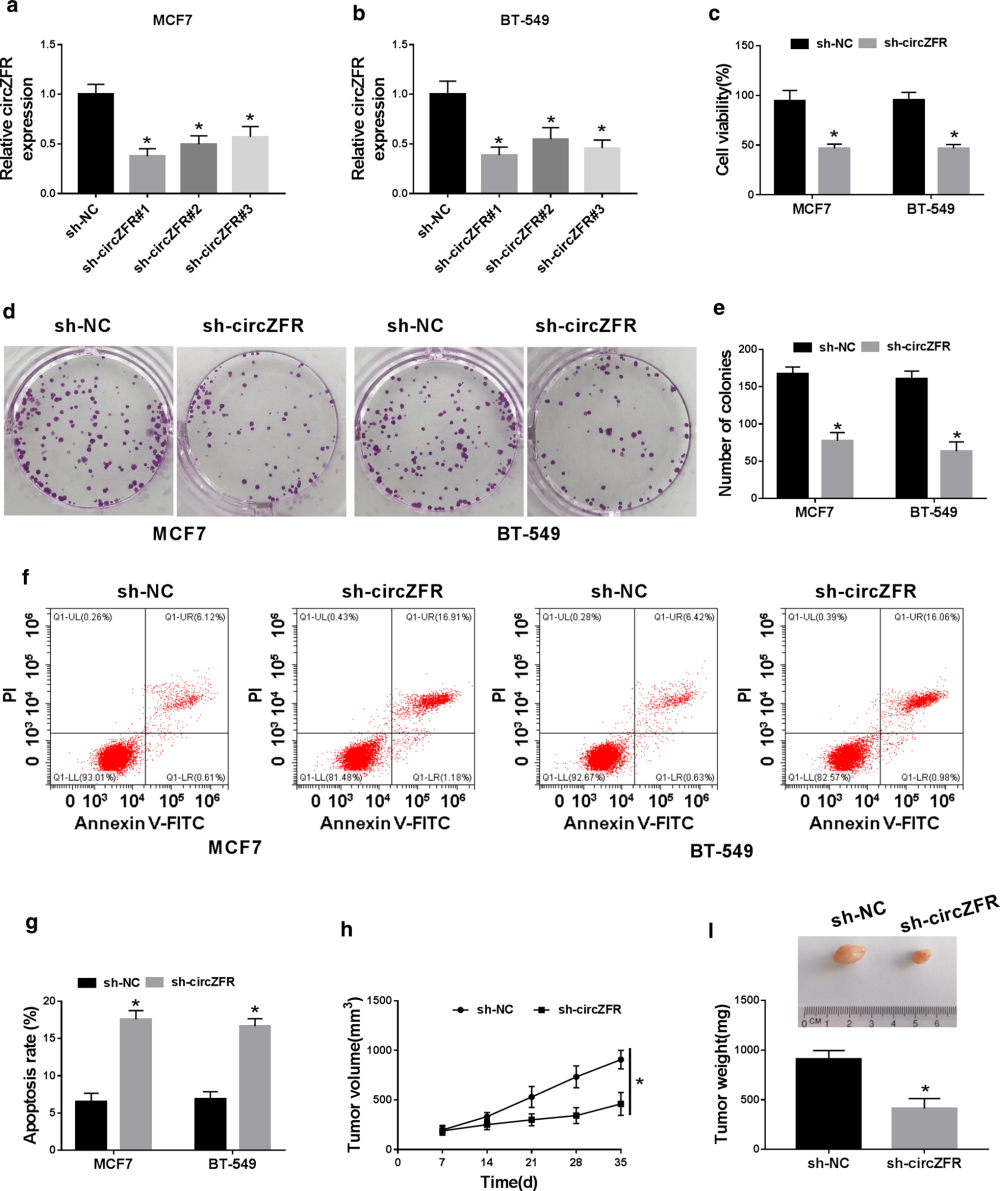
CircZFR silencing suppressed BC progression in vitro and in vivo. **a**, **b** qRT-PCR for circZFR expression in MCF7 and BT-549 cell lines transduced with sh-NC, sh-circZFR#1, sh-circZFR#2 or sh-circZFR#3. CCK-8 assay for cell viability (**c**), colony formation assay for cell colony formation (**d**, **e**), flow cytometry for cell apoptosis (**f**, **g**) in sh-NC-infected or sh-circZFR-transduced MCF7 and BT-549 cell lines. **h**, **i** sh-NC-infected or sh-circZFR-transduced BT-549 cell line was subcutaneously injected into the nude mice (n = 6 per group), followed by the measurement of tumor volume and weight and the capture of representative pictures. *P < 0.05

To study the biological role of circZFR in BC progression, the loss-of-function experiments were carried out using shRNAs against circZFR (sh-circZFR#1, sh-circZFR#2 and sh-circZFR#3). In contrast to the negative control, the transfection of the three shRNAs prominently reduced circZFR expression in both MCF7 and BT-549 cell lines (Fig. 2a, b). Notably, sh-circZFR#1 (also named sh-circZFR) caused the most significant down-regulation in circZFR expression, so we used it for further analyses. Functional experiments data revealed that the silencing of circZFR led to a striking inhibition in cell viability (Fig. 2c), colony formation (Fig. 2d, e), as well as a strong promotion in cell apoptosis (Fig. 2f, g).

To determine whether circZFR regulated BC tumor development in vivo, we performed the xenograft model assays. When we infected BT-549 cell line with sh-circZFR, tumor growth was remarkably blocked compared with the sh-NC control (Fig. 2h, i).

### Silencing of circZFR suppressed BC cell migration, invasion and glycolysis

**Fig. 3:**
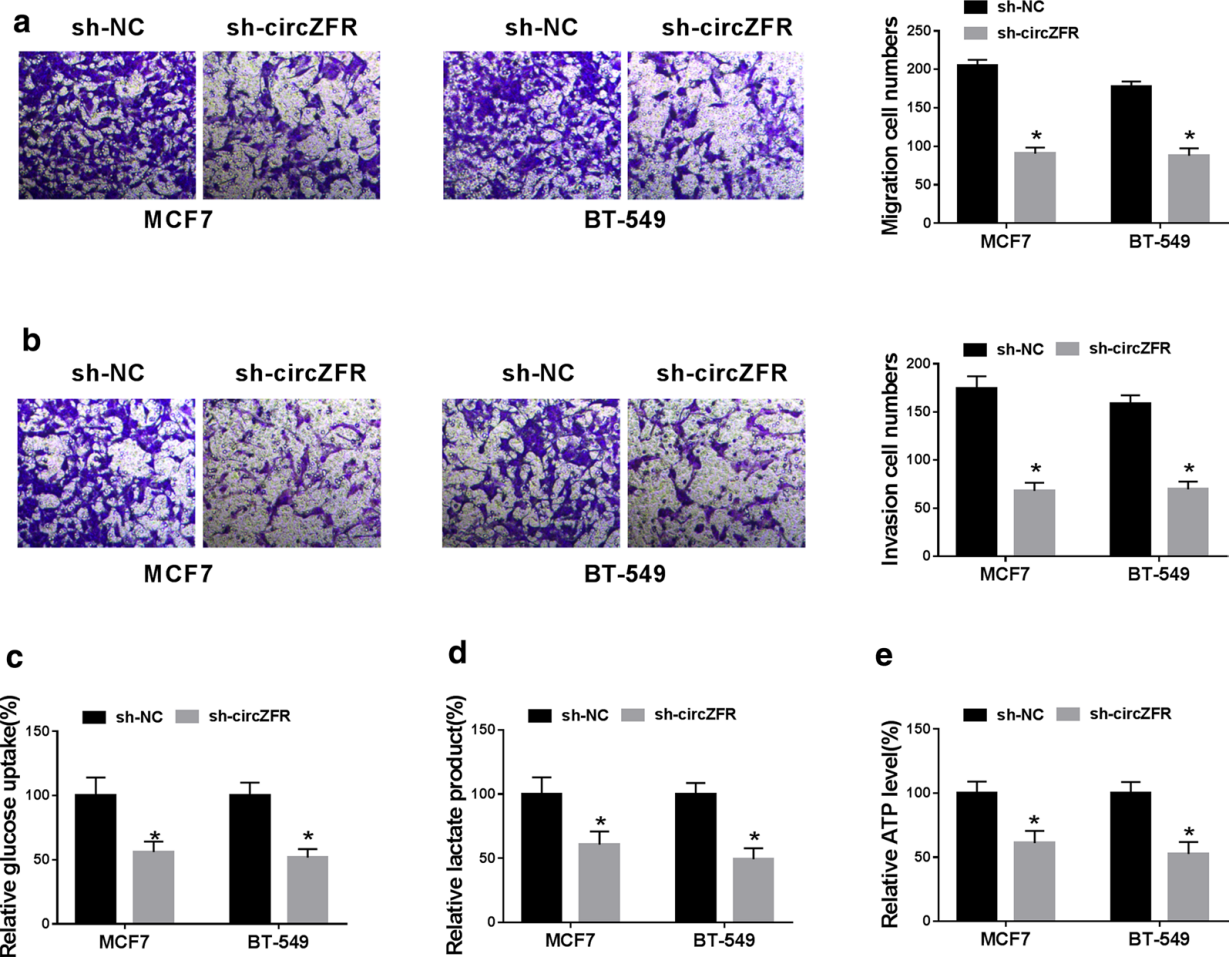
CircZFR silencing suppressed BC cell migration, invasion and glycolysis. Transwell assay for cell migration and invasion (**a**, **b**), corresponding assay kits for glucose uptake, lactate product and ATP level (**c**–**e**) in sh-NC-infected or sh-circZFR-transduced MCF7 and BT-549 cell lines. *P < 0.05

We also asked whether circZFR regulated BC cell migration, invasion and glycolysis in vitro. Transwell assays showed that in comparison to the control group, cell migration (Fig. 3a) and invasion (Fig. 3b) were significantly repressed by circZFR knockdown. Moreover, in both cell lines, circZFR silencing resulted in decreased glucose uptake (Fig. 3c), lactate product (Fig. 3d) and ATP level (Fig. 3e), demonstrating the suppression of circZFR knockdown on cell glycolysis.

### CircZFR directly interacted with miR-578 by binding to miR-578

**Fig. 4:**
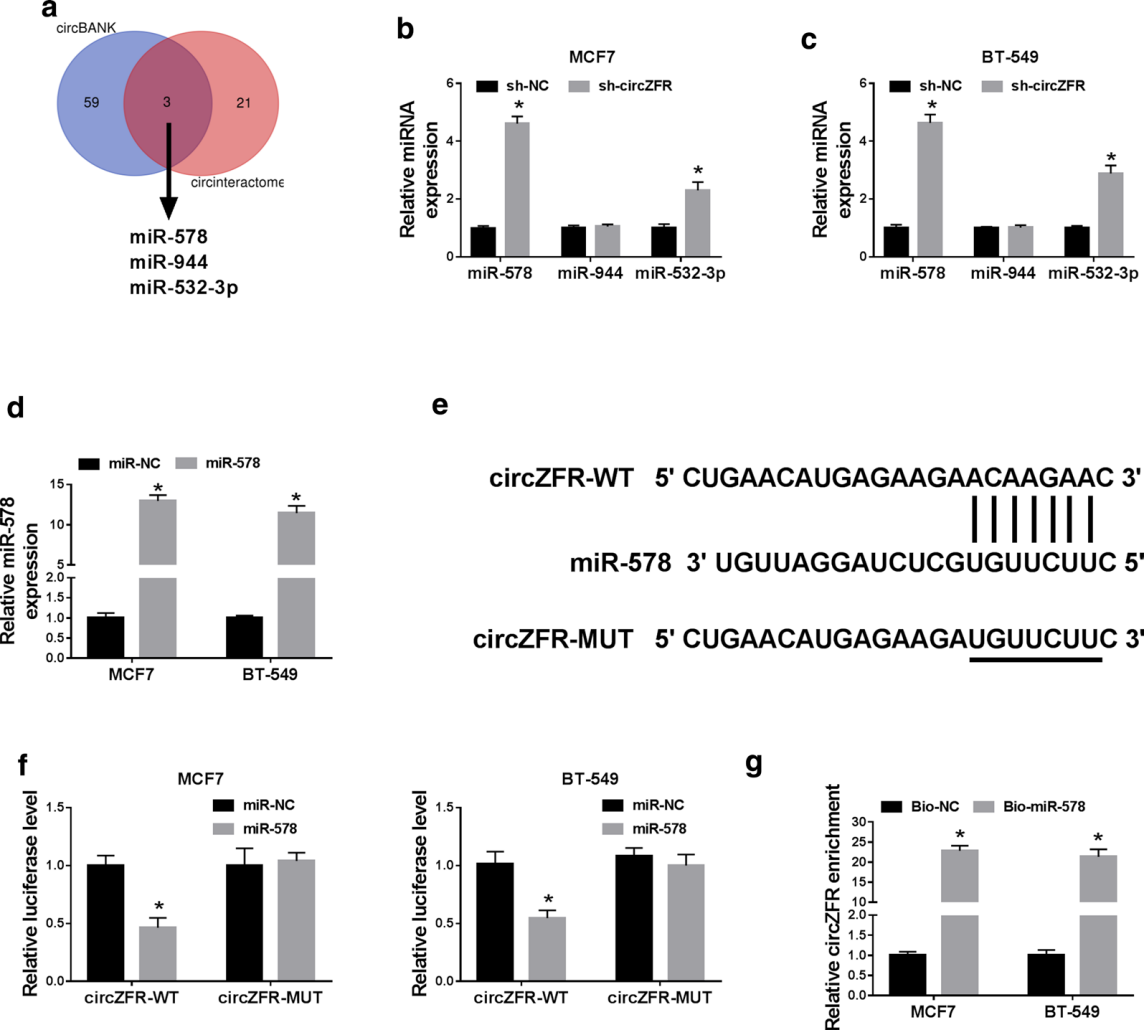
CircZFR directly interacted with miR-578 by binding to miR-578. **a** Venn diagrams representing the putative miRNAs that bind to circZFR identified by CircInteractome and CircBANK online algorithms. **b**, **c** The levels of miR-578, miR-944 and miR-532-3p by qRT-PCR in sh-NC-infected or sh-circZFR-transduced MCF7 and BT-549 cell lines. **d** qRT-PCR for miR-578 expression in the two BC cell lines transfected with miR-NC mimic or miR-578 mimic. **e** Schematic of the miR-578-binding sites within circZFR and the mutation of the seed sequence. **f** Relative luciferase activity in MCF7 and BT-549 cell lines cotransfected with circZFR-WT or circZFR-MUT and miR-NC mimic or miR-578 mimic. **g** qRT-PCR for circZFR level in cell lysates incubated with Bio-NC or Bio-miR-578 and streptavidin beads. *P < 0.05

To further understand the role of circZFR in BC pathogenesis, we performed a detailed analysis for its targeted miRNAs. The two online algorithms CircInteractome and CircBANK collectively revealed that circZFR harbored a putative complementary sequence for miR-578, miR-944 and miR-532-3p (Fig. 4a). The data of qRT-PCR showed that the silencing of circZFR led to a striking overexpression in miR-578 and miR-532-3p levels, but miR-944 expression was not affected by circZFR knockdown in the two BC cell lines (Fig. 4b, c). Previous work had reported that circZFR regulated colorectal cancer progression through acting as a sponge of miR-532-3p [20]. So, we aimed to identify whether miR-578 was a molecular mediator of circZFR in BC progression. By contrast, miR-578 was prominently up-regulated in the two BC cell lines transfected with miR-578 mimic (Fig. 4d). We then cloned circZFR fragment containing the miR-578-binding sites into a luciferase plasmid and mutated the miR-578-binding sites (Fig. 4e). The elevated miR-578 expression significantly reduced the activity of circZFR wild-type reporter (circZFR-WT) (Fig. 4f). However, the mutation of the target sites (circZFR-MUT) completely abrogated the effect of miR-578 on reporter gene expression (Fig. 4f), indicating the validity of the target sequence for interaction. Additionally, RNA pulldown assays revealed that the enrichment level of circZFR was remarkably elevated by Bio-miR-578 in both cell lines (Fig. 4g).

### Overexpression of miR-578 restrained BC cell viability, colony formation, migration, invasion and glycolysis and promoted apoptosis in vitro

**Fig. 5:**
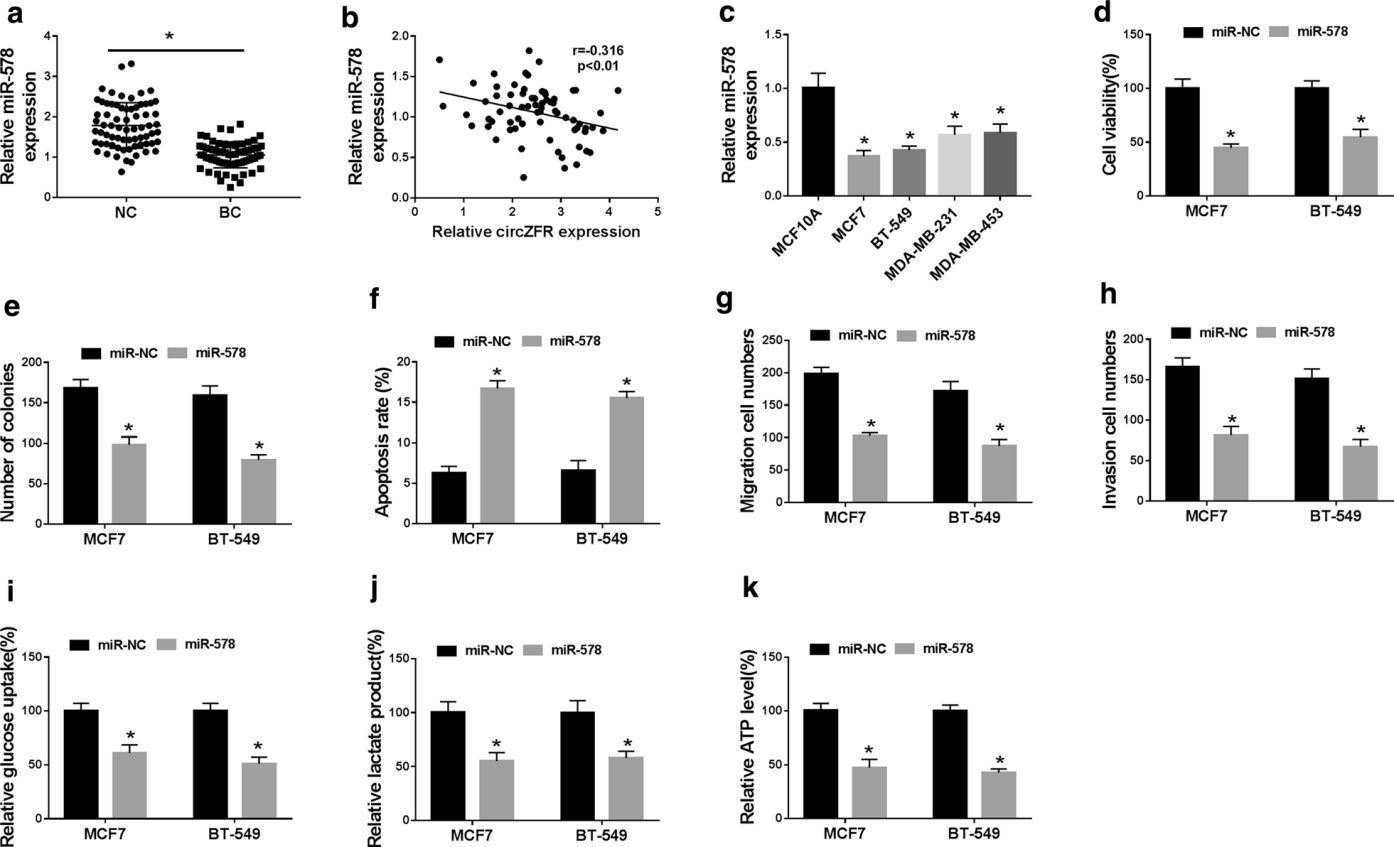
MiR-578 overexpression hindered BC progression in vitro. **a** qRT-PCR for miR-578 expression in 70 pairs of tumor tissues and adjacent normal tissues. **b** Correlation between miR-578 expression and circZFR level in BC tissues using the Spearman test. **c** MiR-578 expression by qRT-PCR in MCF10A, MCF7, BT-549, MDA-MB-231 and MDA-MB-453 cell lines. MCF7 and BT-549 cell lines were transfected with miR-NC mimic or miR-578 mimic, followed by the determination of cell viability by CCK-8 assay (**d**), colony formation using a standard colony formation assay (**e**), cell apoptosis by flow cytometry (**f**), cell migration (**g**) and invasion (**h**) by transwell assay, glucose uptake, lactate product and ATP level using assay kits (**i**–**k**). *P < 0.05

In BC tissues, miR-578 expression was significantly decreased compared to the normal control (Fig. 5a), and it was inversely correlated with circZFR level (Fig. 5b). Moreover, miR-578 level was lower in BC cell lines than that of control (Fig. 5c). Subsequently, we analyzed the biological effect of miR-578 on BC progression. In contrast to the control group, the increased expression of miR-578 induced a distinct repression in cell viability (Fig. 5d) and colony formation (Fig. 5e), and a strong promotion in cell apoptosis (Fig. 5f), as well as a striking reduction in cell migration (Fig. 5G), invasion (Fig. 5H) and glycolysis (Fig. 5i–k).

### Silencing of circZFR regulated BC cell viability, colony formation, migration, invasion, glycolysis and apoptosis in vitro by up-regulating miR-578

**Fig. 6:**
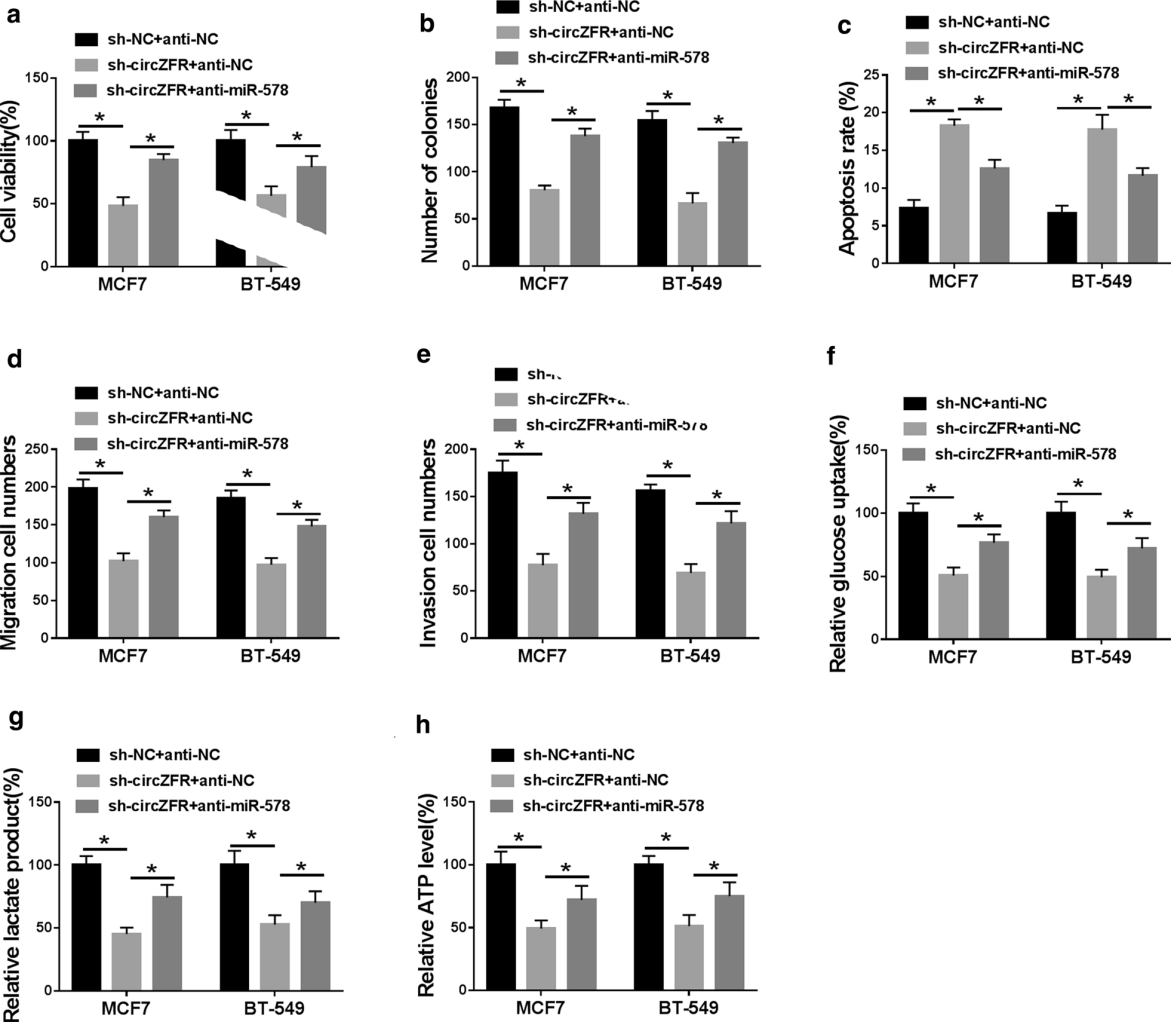
CircZFR knockdown repressed BC malignant progression by miR-578 in vitro. sh-NC-infected or sh-circZFR-transduced MCF7 and BT-549 cell lines were transiently transfected with anti-NC or anti-miR-578. **a** CCK-8 assay for cell viability. **b** A standard colony formation assay for cell colony formation. **c** Flow cytometry for cell apoptosis. **d**, **e** Transwell assay for cell migration and invasion. **f**–**h** Glucose uptake, lactate product and ATP level using assay kits. *P < 0.05

A crucial question was whether circZFR regulated BC progression by miR-578. To address this, we reduced miR-578 expression in sh-circZFR-transduced MCF7 and BT-549 cell lines. As expected, in comparison to the negative control, the reduced level of miR-578 significantly reversed circZFR knockdown-induced anti-viability (Fig. 6a), anti-colony formation (Fig. 6b), pro-apoptosis (Fig. 6c), anti-migration (Fig. 6d), anti-invasion (Fig. 6e) and anti-glycolysis (Fig. 6f–h).

### CircZFR modulated HIF1A expression via acting as a sponge of miR-578

**Fig. 7:**
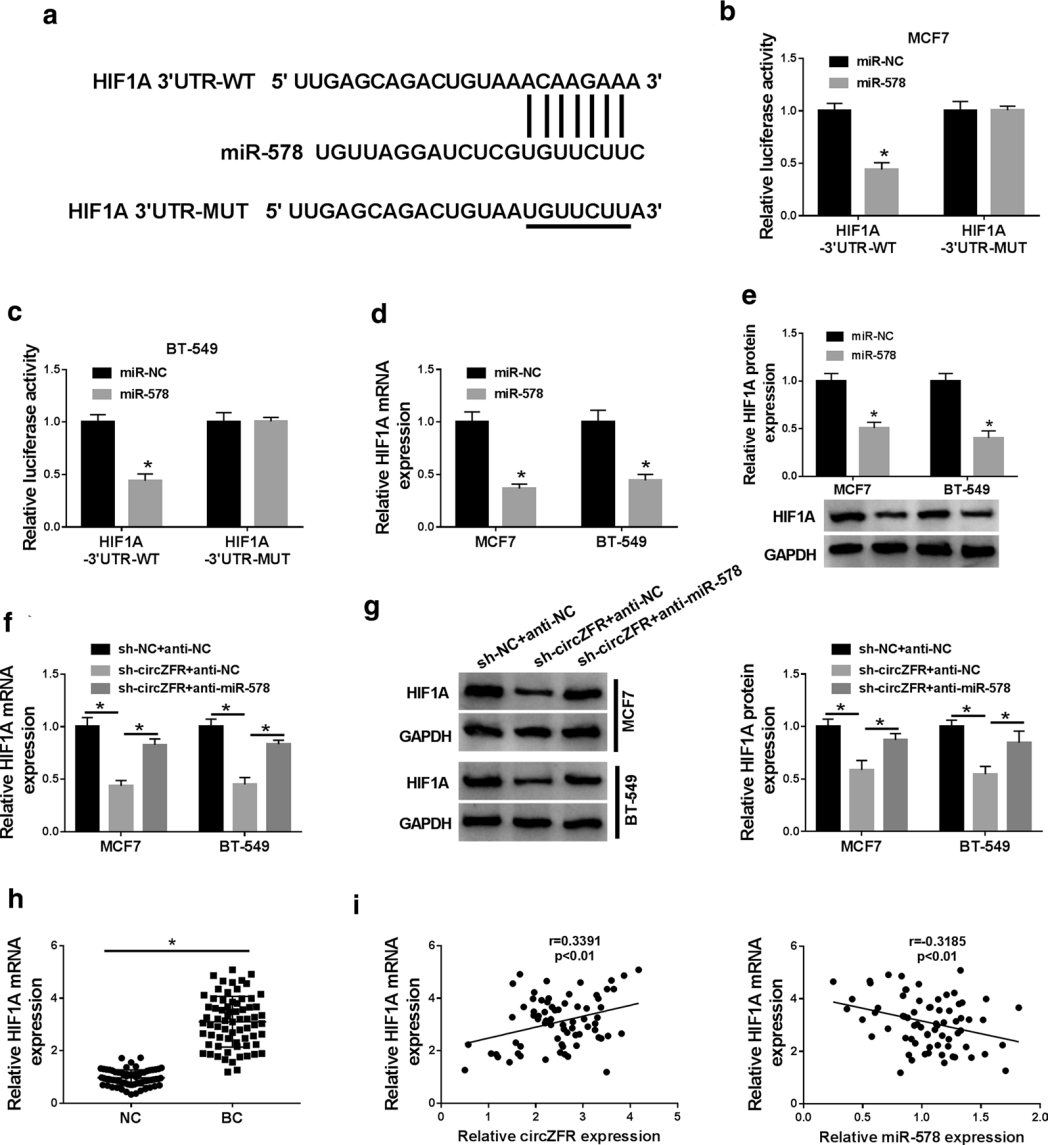
CircZFR modulated HIF1A expression via sponging miR-578. **a** Schematic of the putative miR-578-binding sequence and mutated the target sequence. **b**, **c** Relative luciferase activity in MCF7 and BT-549 cell lines cotransfected with HIF1A-3′UTR-WT or HIF1A-3′UTR-MUT and miR-578 mimic or miR-NC mimic. HIF1A mRNA and protein levels by qRT-PCR and western blot in MCF7 and BT-549 cell lines transfected with miR-578 mimic or miR-NC mimic (**d**, **e**), sh-NC-infected or sh-circZFR-transduced MCF7 and BT-549 cell lines transfected with anti-NC or anti-miR-578 (**f**, **g**). **h** Relative HIF1A mRNA expression by qRT-PCR in 70 pairs of tumor tissues and adjacent normal tissues. **i** Correlation between HIF1A expression with circZFR or miR-578 level in BC tissues using Spearman test. *P < 0.05

Using the software TargetScan, a predicted miR-578-binding sequence (ACAAGAA) was identified within the 3′UTR of HIF1A (Fig. 6a). When we cloned the 3′UTR fragment containing the putative miR-578-binding sites downstream of a luciferase coding sequence, the cotransfection of the luciferase reporter (HIF1A-3′UTR-WT) and miR-578 mimic into the two BC cell lines produced lower luciferase activity than in cell lines cotransfected with the miR-NC control (Fig. 7b, c). However, the mutation of the target sequence (HIF1A-3′UTR-MUT) prominently abolished the suppression of miR-578 (Fig. 7b, c). By contrast, HIF1A mRNA and protein levels were significantly reduced by miR-578 overexpression in the two BC cell lines (Fig. 7d, e). These data together pointed that HIF1A in BC cell lines was directly targeted and inhibited by miR-578.

We then examine whether circZFR influenced HIF1A expression in BC cell lines. As expected, in comparison to their counterparts, HIF1A expression was remarkably down-regulated by circZFR silencing at both mRNA and protein levels in the two BC cell lines, and this effect was significantly abrogated by anti-miR-578 introduction (Fig. 7f, g). Additionally, qRT-PCR data showed a striking up-regulation of HIF1A mRNA level in BC tissues (Fig. 7h). Furthermore, in BC tissues, HIF1A mRNA expression was positively correlated with circZFR expression and negatively correlated with miR-578 level (Fig. 7i).

### HIF1A was a functional target of miR-578 in regulating BC cell viability, colony formation, migration, invasion, glycolysis and apoptosis in vitro

**Fig. 8:**
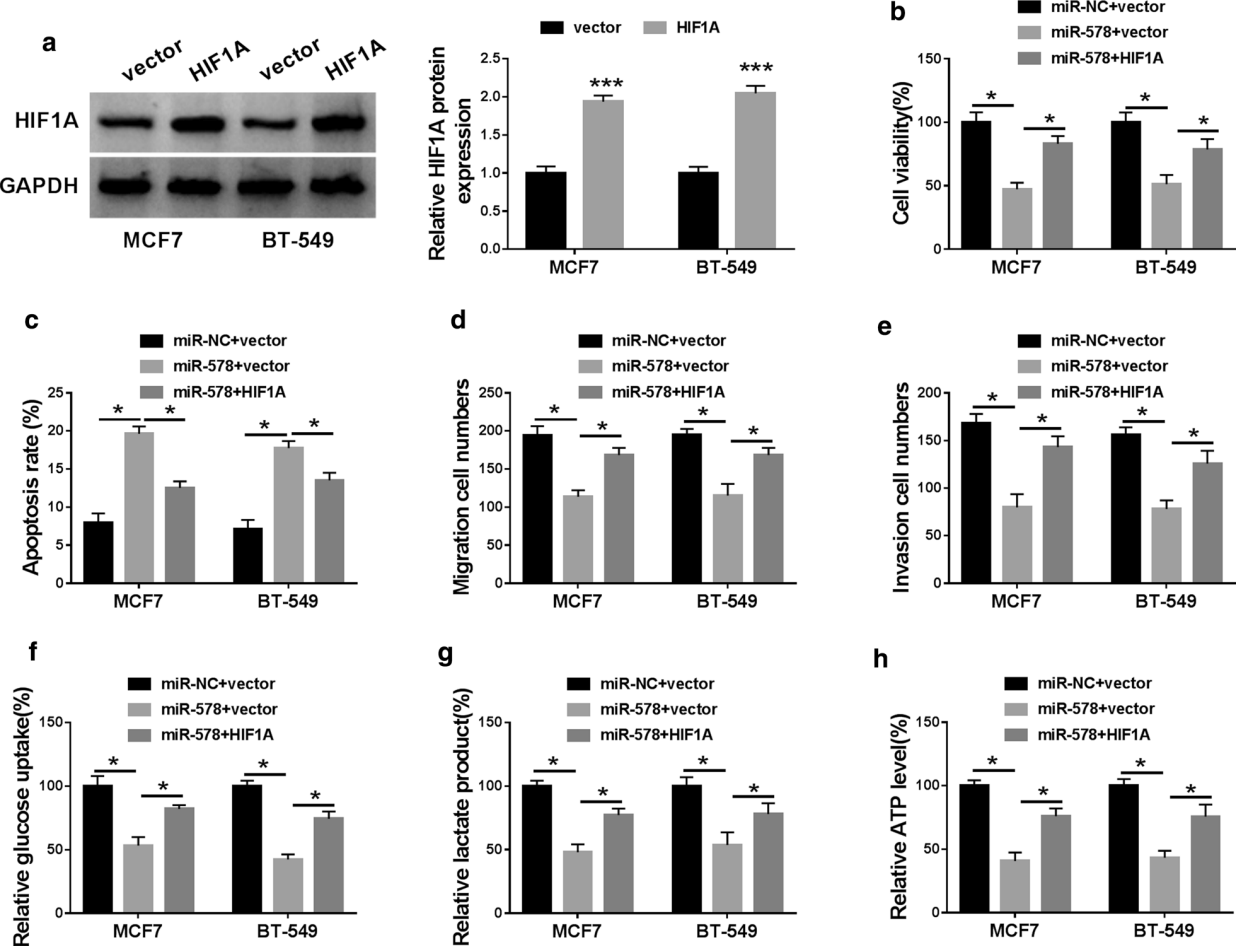
The repression of miR-578 up-regulation on BC progression in vitro was mediated by HIF1A. **a** HIF1A protein level by western blot in MCF7 and BT-549 cell lines transfected with vector or HIF1A. MCF7 and BT-549 cell lines were transfected with miR-NC mimic + vector, miR-578 mimic + vector or miR-578 mimic + HIF1A, followed by the determination of cell viability by CCK-8 assay (**b**), cell apoptosis by flow cytometry (**c**), cell migration and invasion by transwell assay (**d**, **e**), glucose uptake, lactate product and ATP level using assay kits (**f**–**h**). vector: negative control plasmid, HIF1A: recombinant HIF1A overexpressing plasmid. *P < 0.05

To provide further insight into the link between miR-578 and HIF1A in BC progression, we elevated HIF1A expression using a recombinant overexpressing plasmid in miR-578 mimic-transfected BC cell lines. As a result, HIF1A protein level was prominently increased by the overexpressing plasmid in the two cell lines (Fig. 8a). Functional experiments results revealed that the up-regulation of HIF1A dramatically abolished miR-578 overexpression-mediated anti-viability (Fig. 8b), pro-apoptosis (Fig. 8c), anti-migration (Fig. 8d), anti-invasion (Fig. 8e) and anti-glycolysis (Fig. 8f–h).

## Discussion

To date, the emerging links between circRNAs and BC progression have opened up a novel field for cancer diagnosis and treatment [21, 22]. In the meantime, understanding the molecular basis underlying the actions of circRNAs has been still challenging. In this study, we identified the biological role of circZFR in BC progression in vitro and in vivo and investigated the mechanisms governing it.

Here, we firstly demonstrated that circZFR was overexpressed in BC, and the elevated expression of circZFR was associated with the poor prognosis of these patients. By using loss-of-function in vitro and in vivo analyses, we were first to uncover that the silencing of circZFR performed a suppressive effect in BC progression. Previous reports had highlighted the potential oncogenic role of circZFR in several other malignancies, such as hepatocellular carcinoma, papillary thyroid cancer and non-small cell lung cancer [14, 23, 24]. Conversely, circZFR was reported as a tumor suppressor in colorectal cancer and gastric cancer [20, 25]. These contradictory findings may attribute to different type tumor or complex tumor microenvironment.

Using the online algorithms, we first identified that circZFR sequestered miR-578 through sponging miR-578 in BC cell lines. Ji et al. showed that the increased miR-578 level weakened osteosarcoma progression via directly interacting with circ_001621 [26]. Farhana et al. unraveled that in pancreatic cancer, miR-578 was associated with the tumor cell apoptosis [27]. As it has been reported [16], our data validated the down-regulation of miR-578 expression in BC tissues and cells. Moreover, we first identified that miR-578 overexpression restrained BC cell malignant behaviors in vitro. More importantly, for the first time, we substantiated that circZFR knockdown hampered BC progression in vitro by miR-578.

Using TargetScan software, we identified that miR-578 in BC cell lines directly targeted and inhibited HIF1A. HIF1A, a transcriptional regulator in response to intratumoral hypoxia [28, 29], contributes to BC metastasis and malignant progression [30–32]. We also uncovered that the up-regulation of miR-578 hampered BC malignant progression via down-regulating HIF1A in vitro. Previous studies had reported that several other miRNAs, such as miR-18a and miR-497, exerted an anti-tumor activity in BC through targeting HIF1A [33, 34]. Furthermore, our data first illuminated the role of circZFR as a sponge of miR-578 to mediate HIF1A expression in BC cell lines. The findings by Liang et al. underscored that circRNA circDENND4C contributed to BC cell proliferation under hypoxia via regulating HIF1A [35].

In conclusion, our present study demonstrated that circZFR was overexpressed in BC, and the silencing of circZFR suppressed BC malignant progression via the regulation of the miR-578/HIF1A axis. This is the first report of circZFR in BC pathogenesis, providing evidence for the crucial involvement of circZFR in BC progression.

## Supplementary information

**Additional file 1: Supplement material 1.** The STR authentication of MCF7 (A) and BT-549 (B) cells.

**Additional file 2: Supplement material 2.** The detailed quantification of the western blot analysis.

## Data Availability

The datasets used and/or analysed during the current study are available from the corresponding author on reasonable request (Additional files 1, 2).

## Abbreviations

BC: breast cancer
HIF1A: hypoxia-inducible factor 1α
CCK-8: Cell Counting Kit-8
ATP: adenosine triphosphate
ANOVA: analysis of variance

## References

[1] Bray F, Ferlay J, Soerjomataram I, Siegel RL, Torre LA, Jemal A (2018). Global cancer statistics 2018: GLOBOCAN estimates of incidence and mortality worldwide for 36 cancers in 185 countries. CA Cancer J Clin.

[2] Wörmann B (2017). Breast cancer: basics, screening, diagnostics and treatment. Med Monatsschr Pharm.

[3] Arnedos M, Vicier C, Loi S, Lefebvre C, Michiels S, Bonnefoi H (2015). Precision medicine for metastatic breast cancer–limitations and solutions. Nat Rev Clin Oncol..

[4] Kristensen LS, Andersen MS, Stagsted LVW, Ebbesen KK, Hansen TB (2019). The biogenesis, biology and characterization of circular RNAs. Nat Rev Genet.

[5] Denzler R, Agarwal V, Stefano J, Bartel DP, Stoffel M (2014). Assessing the ceRNA hypothesis with quantitative measurements of miRNA and target abundance. Mol Cell.

[6] Wang X, Fang L (2018). Advances in circular RNAs and their roles in breast Cancer. J Exp Clin Cancer Res..

[7] Yuan P, Lei L, Dong S, Liu D (2020). Circular RNA hsa_circ_0068033 acts as a diagnostic biomarker and suppresses the progression of breast cancer through sponging miR-659. Onco Targets Ther..

[8] Cao L, Wang M, Dong Y, Xu B, Chen J, Ding Y, Zaharieva E, Zhou X (2020). Circular RNA circRNF20 promotes breast cancer tumorigenesis and Warburg effect through miR-487a/HIF-1α/HK2. Cell Death Dis..

[9] Xu JZ, Shao CC, Wang XJ, Zhao X, Chen JQ, Ouyang YX (2019). circTADA2As suppress breast cancer progression and metastasis via targeting miR-203a-3p/SOCS3 axis. Cell Death Dis..

[10] Liu Z, Zhou Y, Liang G, Ling Y, Tan W, Tan L (2019). Circular RNA hsa_circ_001783 regulates breast cancer progression via sponging miR-200c-3p. Cell Death Dis..

[11] Liang HF, Zhang XZ, Liu BG, Jia GT, Li WL (2017). Circular RNA circ-ABCB10 promotes breast cancer proliferation and progression through sponging miR-1271. Am J Cancer Res..

[12] Wang M, Gao Y, Liu J (2019). Silencing circZFR inhibits the proliferation, migration and invasion of human renal carcinoma cells by regulating miR-206. Onco Targets Ther..

[13] Zhang WY, Liu QH, Wang TJ, Zhao J, Cheng XH, Wang JS (2019). CircZFR serves as a prognostic marker to promote bladder cancer progression by regulating miR-377/ZEB2 signaling. Biosci Rep..

[14] Zhang H, Wang X, Hu B, Zhang F, Wei H, Li L (2019). Circular RNA ZFR accelerates non-small cell lung cancer progression by acting as a miR-101-3p sponge to enhance CUL4B expression. Artif Cells Nanomed Biotechnol..

[15] Asiaf A, Ahmad ST, Arjumand W, Zargar MA (2018). MicroRNAs in breast cancer: diagnostic and therapeutic potential. Methods Mol Biol.

[16] Danza K, De Summa S, Pinto R, Pilato B, Palumbo O, Merla G (2015). MiR-578 and miR-573 as potential players in BRCA-related breast cancer angiogenesis. Oncotarget..

[17] Kanaoka R, Iinuma H, Dejima H, Sakai T, Uehara H, Matsutani N (2018). Usefulness of plasma exosomal MicroRNA-451a as a noninvasive biomarker for early prediction of recurrence and prognosis of non-small cell lung cancer. Oncology..

[18] Ding C, Yi X, Wu X, Bu X, Wang D, Wu Z (2020). Exosome-mediated transfer of circRNA CircNFIX enhances temozolomide resistance in glioma. Cancer Lett.

[19] Zhao C, Wang W, Yu W, Jou D, Wang Y, Ma H (2016). A novel small molecule STAT3 inhibitor, LY5, inhibits cell viability, colony formation, and migration of colon and liver cancer cells. Oncotarget..

[20] Bian L, Zhi X, Ma L, Zhang J, Chen P, Sun S (2018). Hsa_circRNA_103809 regulated the cell proliferation and migration in colorectal cancer via miR-532-3p/FOXO4 axis. Biochem Biophys Res Commun.

[21] Zhang HD, Jiang LH, Sun DW, Hou JC, Ji ZL (2018). CircRNA: a novel type of biomarker for cancer. Breast Cancer..

[22] Klinge CM. Non-Coding RNAs in breast cancer: intracellular and intercellular communication. Non-coding RNA. 2018;4(4). 10.3390/ncrna4040040 PMC6316884 30545127

[23] Tan A, Li Q, Chen L (2019). CircZFR promotes hepatocellular carcinoma progression through regulating miR-3619-5p/CTNNB1 axis and activating Wnt/β-catenin pathway. Arch Biochem Biophys.

[24] Wei H, Pan L, Tao D, Li R (2018). Circular RNA circZFR contributes to papillary thyroid cancer cell proliferation and invasion by sponging miR-1261 and facilitating C8orf4 expression. Biochem Biophys Res Commun.

[25] Liu T, Liu S, Xu Y, Shu R, Wang F, Chen C (2018). Circular RNA-ZFR inhibited cell proliferation and promoted apoptosis in gastric cancer by sponging miR-130a/miR-107 and modulating PTEN. Cancer Res Treat..

[26] Ji X, Shan L, Shen P, He M (2020). Circular RNA circ_001621 promotes osteosarcoma cells proliferation and migration by sponging miR-578 and regulating VEGF expression. Cell Death Dis..

[27] Farhana L, Dawson MI, Fontana JA (2015). Down regulation of miR-202 modulates Mxd1 and Sin3A repressor complexes to induce apoptosis of pancreatic cancer cells. Cancer Biol Ther.

[28] Hayashi Y, Yokota A, Harada H, Huang G (2019). Hypoxia/pseudohypoxia-mediated activation of hypoxia-inducible factor-1α in cancer. Cancer Sci.

[29] Akanji MA, Rotimi D, Adeyemi OS (2019). Hypoxia-inducible factors as an alternative source of treatment strategy for cancer. Oxid Med Cell Longev..

[30] De Francesco EM, Maggiolini M (2018). Crosstalk between Notch, HIF-1α and GPER in breast cancer EMT. Int J Mol Sci.

[31] Ponente M, Campanini L, Cuttano R, Piunti A, Delledonne GA, Coltella N (2017). PML promotes metastasis of triple-negative breast cancer through transcriptional regulation of HIF1A target genes. JCI Insight..

[32] Tosatto A, Sommaggio R, Kummerow C, Bentham RB, Blacker TS, Berecz T (2016). The mitochondrial calcium uniporter regulates breast cancer progression via HIF-1α. EMBO Mol Med..

[33] Krutilina R, Sun W, Sethuraman A, Brown M, Seagroves TN, Pfeffer LM (2014). MicroRNA-18a inhibits hypoxia-inducible factor 1α activity and lung metastasis in basal breast cancers. Breast Cancer Res.

[34] Wu Z, Cai X, Huang C, Xu J, Liu A (2016). miR-497 suppresses angiogenesis in breast carcinoma by targeting HIF-1α. Oncol Rep.

[35] Liang G, Liu Z, Tan L, Su AN, Jiang WG, Gong C (2017). HIF1α-associated circDENND4C promotes proliferation of breast cancer cells in hypoxic environment. Anticancer Res.

