# Supplementary figures and images for: CircZFR functions as a sponge of miR-578 to promote breast cancer progression by regulating HIF1A expression
Source: Cancer Cell Int. 2020 Aug 18;20:400. doi: 10.1186/s12935-020-01492-5 (PMC7437024; doi:10.1186/s12935-020-01492-5)

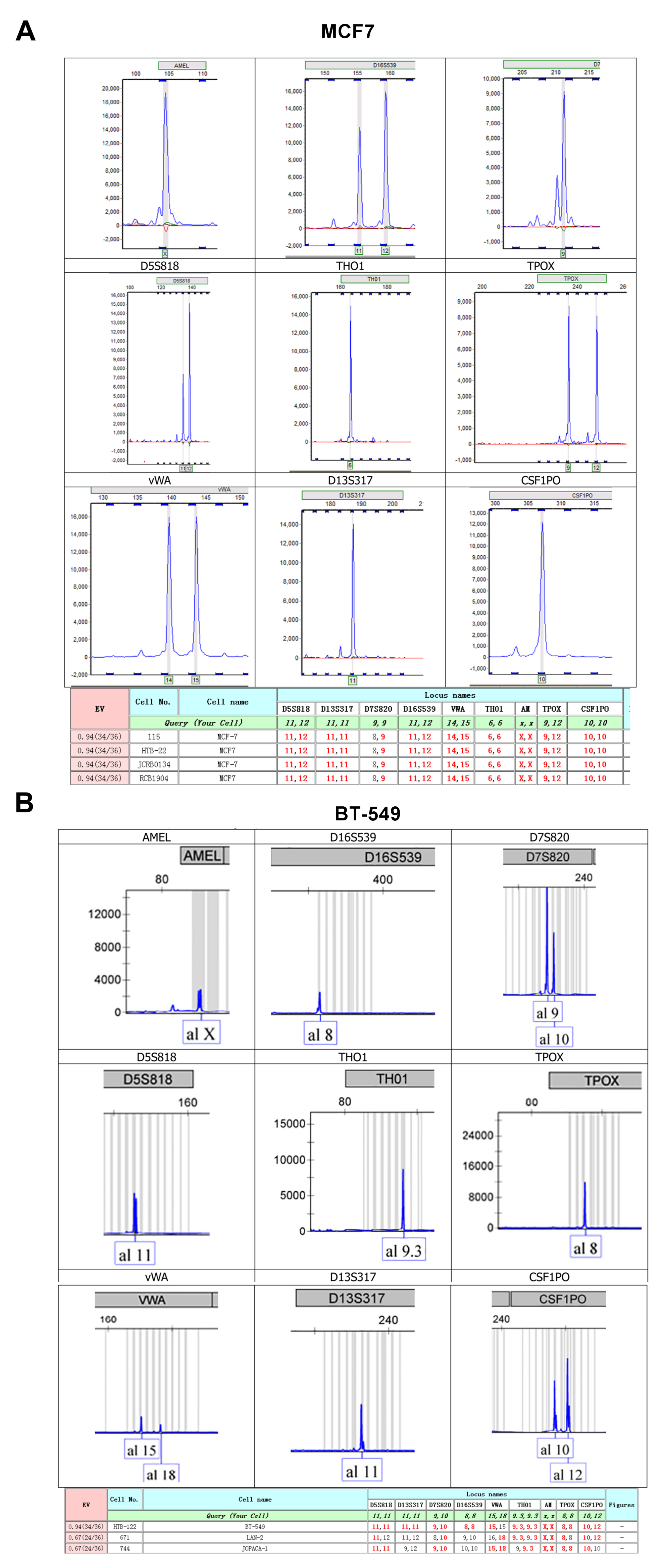

Supplement: Supplementary file 1 — Additional file 1: Supplement material 1. The STR authentication of MCF7 (A) and BT-549 (B) cells. [file 12935_2020_1492_MOESM1_ESM.tif]
